# Mobile Health Strategies to Tackle Skin Neglected Tropical Diseases With Recommendations From Innovative Experiences: Systematic Review

**DOI:** 10.2196/22478

**Published:** 2020-12-31

**Authors:** Carme Carrion, Noemí Robles, Oriol Sola-Morales, Marta Aymerich, Jose Antonio Ruiz Postigo

**Affiliations:** 1 eHealth Lab Research Group School of Health Sciences Universitat Oberta de Catalunya Barcelona Spain; 2 eHealth Center Universitat Oberta de Catalunya Barcelona Spain; 3 Red de Investigación en Servicios Sanitarios en Enfermedades Crónicas (REDISSEC) Barcelona Spain; 4 Health Innovation Technology Transfer Barcelona, Catalonia Spain; 5 Prevention, Treatment and Care Unit Department of Control of Neglected Tropical Diseases World Health Organization Geneve Switzerland

**Keywords:** mHealth, mobile health, neglected tropical diseases, skin neglected tropical diseases, apps, SMS text messaging, low- and middle-income countries

## Abstract

**Background:**

Neglected tropical diseases (NTDs) represent a diverse group of 20 communicable diseases that occur in tropical and subtropical areas in 149 countries, affecting over 1 billion people and costing developing economies billions of dollars every year. Within these diseases, those that present lesions on the skin surface are classified as skin NTDs (sNTDs). Mobile health interventions are currently being used worldwide to manage skin diseases and can be a good strategy in the epidemiological and clinical management of sNTDs.

**Objective:**

We aimed to analyze existing evidence about mobile health interventions to control and manage sNTDs in low- and middle-income countries (LMICs) and make recommendations for what should be considered in future interventions.

**Methods:**

A systematic review was conducted of the MEDLINE, Embase, and Scopus databases over 10 years up to April 30, 2020. All types of clinical studies were considered. Data were synthesized into evidence tables. Apps were selected through a comprehensive systematic search in the Google Play Store and Apple App Store conducted between March 20 and April 15, 2020.

**Results:**

From 133 potentially relevant publications, 13 studies met our criteria (9.8%). These analyzed eight different interventions (three SMS text messaging interventions and five app interventions). Six of the 13 (46%) studies were community-based cross-sectional studies intended to epidemiologically map a specific disease, mainly lymphatic filariasis, but also cutaneous leishmaniasis, leprosy, and NTDs, as well as sNTDs in general. Most of the studies were considered to have a high (5/13, 39%) or moderate (4/13, 31%) risk of bias. Fifteen apps were identified in the Google Play Store, of which three were also in the Apple App Store. Most of the apps (11/15, 73%) were targeted at health care professionals, with only four targeted at patients. The apps focused on scabies (3/15, 20%), lymphatic filariasis (3/15, 20%), cutaneous leishmaniasis (1/15, 7%), leprosy (1/15, 7%), yaws and Buruli ulcer (1/15, 7%), tropical diseases including more than one sNTDs (3/15, 20%), and NTDs including sNTDs (2/15, 13%). Only 1 (7%) app focused on the clinical management of sNTDs.

**Conclusions:**

All mobile health interventions that were identified face technological, legal, final user, and organizational issues. There was a remarkable heterogeneity among studies, and the majority had methodological limitations that leave considerable room for improvement. Based on existing evidence, eight recommendations have been made for future interventions.

## Introduction

According to the World Health Organization (WHO), neglected tropical diseases (NTDs) represent a diverse group of 20 communicable diseases that occur in tropical and subtropical conditions in 149 countries, affecting over 1 billion people and costing developing economies billions of dollars every year [1]. These diseases include protozoal, bacterial, helminth, and viral infections [2] that cause vast suffering, stigma, and disability, and frequently lead to death [3]. As a result, NTDs trap impoverished people in a cycle of poverty and disease. Collectively, NTDs are among the most devastating of communicable diseases in terms of not only the global health burden (26.1 million disability-adjusted life-years) [3], but also the impact on development and overall economic productivity in low- and middle-income countries (LMICs).

The WHO has further categorized NTDs that primarily present as lesions on the skin (lumps or swelling, ulcers, swollen limbs, and patches on the face or body) into the skin NTD (sNTD) group. This includes Buruli ulcers, cutaneous leishmaniasis, post-kala-azar dermal leishmaniasis, leprosy, lymphatic filariasis (lymphoedema and hydrocele), mycetoma, onchocerciasis, fungal infections, scabies, and yaws. sNTDs not only cause considerable disability and stigma, but also exacerbate poverty [4-6]. The integration of mapping, surveillance, clinical diagnosis, and management has only been achieved in a limited range of settings and disease groupings [7]. Unfortunately, there has been relatively little investment in laboratory research, epidemiology, diagnostic tools, and management strategies to control tropical skin diseases. Visual examination provides an opportunity to screen people in communities or children in schools to identify multiple conditions in a single visit. This common approach to skin diseases justifies the integrated delivery of health care interventions to both increase cost-effectiveness and expand coverage [8,9].

Various innovative methods have been used to enhance the clinical management and epidemiological surveillance of, among others, skin and infectious diseases worldwide. These include, but are not limited to, technological methods such as telemedicine, artificial intelligence, and mobile health (mHealth) [10]. mHealth has been proven to be a promising tool to improve the diagnosis and treatment of several diseases such as skin cancer [11]. Telemedicine interventions have been implemented to perform teleconsultation and to improve the diagnosis and treatment of several diseases in countries with poor resources, and they have been proven to be effective in achieving its goal [12,13]. Teledermatological interventions, consisting of real-time videoconferencing or asynchronous transition of images between different dermatologists or between patients and clinicians, have been used for some years. Numerous studies have been published describing the potential of teledermatology in low-income settings, such as the review performed by Médecins Sans Frontières to assess the tele-expertise system it had implemented to improve access to specialized clinical support for its field health workers [14]. These interventions are not yet scaled up, and although trials seem to confirm they are effective, they show some limitations that should be considered, such as the need for both a dermatologist available on demand and a stable and strong internet connection [15]. Artificial intelligence also holds promise in the diagnosis of skin conditions to a good degree of accuracy [16] and selecting the best treatment for a specific infectious disease [17]. However, these techniques are still under development and not yet available for implementation in the clinical management of sNTDs [18].

The WHO Global Observatory for eHealth defines mHealth as “medical and public health practice supported by mobile devices, such as mobile phones, patient monitoring devices, personal digital assistants, and other wireless devices” [19]. The prevention and management of chronic diseases through mHealth strategies has increased over recent years, mainly in high-income countries. However, there is still a lack of evidence for its efficacy, effectiveness, and safety [20]. Attempts have been made to improve the effective surveillance and control of infectious disease outbreaks [21], but very few interventions have been implemented in LMICs where sNTDs are endemic.

Given the high number of smartphone users worldwide (around 3 billion) [22] and the high penetration of smartphones in groups with low socioeconomic status, health-related mobile apps provide an opportunity to overcome traditional barriers to the control and clinical management of sNTDs in LMICs [23,24]. Nevertheless, the vast increase in low-cost health-related apps that are not regulated by health care policymakers raises important areas of concern, including quality, usability, and the need to educate consumers regarding the potentially beneficial (or harmful) content of apps [25]. Currently, of the over 325,000 health-related apps available [26], over 500 are skin related, with 90 providing self-surveillance and diagnosis [27]. Evaluation of six apps for skin cancer diagnosis has been conducted in high-income countries with discouraging results [28], and content analysis of 123 apps demonstrated that all were in need of improvement [29].

Therefore, it is clear that research on implementing mHealth strategies is required to improve the epidemiological surveillance and clinical management of sNTDs in LMICs. The main objective of this article is to analyze existing evidence about mHealth interventions to control and manage sNTDs in LMICs and make recommendations of what should be considered in future interventions.

## Methods

### Systematic Review

#### Information Sources

**Figure 1:**
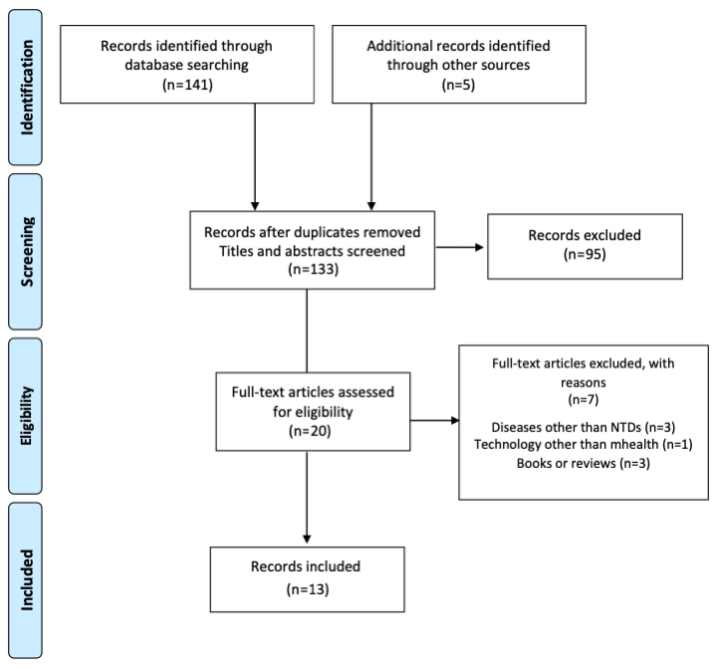
PRISMA flow diagram of the selection of papers for inclusion in the review. NTD: neglected tropical disease.

Searches were conducted in the following databases: MEDLINE, Embase, and Scopus. This was complemented with a manual search of references in key journal archives. All published articles in the 10 years up to April 30, 2020, were considered with no restrictions on language. The reference lists of all selected studies were cross-checked for additional reports. A flow diagram of papers selected has been reported according to the Preferred Reporting Items for Systematic Reviews and Meta-Analyses (PRISMA) statement [30] (Figure 1).

#### Search Strategy

The search strategy included both controlled vocabulary and free-text terms. The terms were smartphone, neglected tropical diseases, Buruli ulcer, fungal disease, leishmaniasis, leprosy, lymphatic filariasis, lymphoedema, mycetoma, onchocerciasis, scabies, yaws, mHealth, SMS, and apps (Multimedia Appendix 1).

#### Eligibility Criteria

The criteria for inclusion were reporting on mHealth diagnoses, treatment, epidemiological surveillance, and prevention of sNTDs. All trial designs, regardless of risk of bias, were considered eligible. Animal studies were excluded. Letters, editorials, protocols, and reviews were not included.

#### Study Selection and Data Collection Process

All identified references were imported into Mendeley (v1.18), and duplicate references were eliminated. Articles that met the inclusion criteria were full-text reviewed by two independent reviewers. Any disagreements were resolved by a third reviewer. Study features and outcomes were entered into a database specifically designed for this review. Risk of bias was assessed according to Scottish Intercollegiate Guidelines Network (SIGN) codes for study assessment [31].

### App Selection

#### Search Strategy

Apps were identified through a comprehensive systematic search of the Google Play Store and Apple App Store between March 20 and April 15, 2020, using 14 search terms in English and Spanish across all store categories (Multimedia Appendix 1).

#### Screening Procedure

Each term was searched separately in both app stores, with two researchers performing identical independent searches. Information in the app store description for each result was reviewed. The inclusion criteria were as follows: (1) the purpose should be diagnosis, treatment, prevention, patient empowerment, health literacy, or epidemiological surveillance; (2) the main disease should be one of the 10 sNTDs; and (3) the app should be available in English or Spanish. Apps were excluded if they did not have a clear focus on the epidemiological or clinical management of sNTDs and focused on other issues, such as conferences, atlases of skin diseases, and a general approach to tropical diseases. Apps that met the eligibility criteria were selected and their URLs were saved. After the independent search, the two researchers compared the lists of apps they identified as eligible and performed analyses. Light and early versions of the apps were excluded in favor of full late versions.

#### Data Collection

Information and features were extracted from app store descriptions and available screenshots. In cases where not enough information was provided, the apps were downloaded, installed, and tested in smartphones running Android or iOS operating system.

## Results

### Selection of Studies

A total of 133 potentially relevant publications were identified as eligible. After screening the titles and abstracts, 20 out of 133 (15.0%) were accepted for full-text review. Out of these 20, 7 (35%) were excluded for not meeting the inclusion criteria. They focused on diseases other than sNTDs (n=3), used interventions without any mHealth element (n=1), or were reviews or book chapters (n=3). After peer-review, 13 articles were included in this nonquantitative review (Figure 1).

The main characteristics of the 13 studies included are detailed in Table 1. Studies appear in chronological order and then alphabetical order of the first author. The studies were highly heterogeneous in their design, resulting from their heterogeneous objectives. Six (46%) studies [32-37] were reported to be community-based cross-sectional studies with the main objective of epidemiologically mapping a specific disease based on its clinical symptoms. Four out of 13 (31%) studies had different designs as follows: a mixed-methods approach to define challenges to be considered [38]; different implementation scenarios for a specific mHealth intervention [39]; the sustainability of an intervention [40]; and the process of developing a specific mHealth tool or assessing its usability [10,38]. One trial was a cross-sectional study analyzing the accuracy of a new diagnosis strategy [41], and another was a prospective cohort study testing the efficacy of an mHealth strategy in training health care workers [42]. There was only one controlled nonrandomized study to test the effectiveness of an intervention [43]. Most of the studies had a high (5/13, 39%) or moderate (4/13, 31%) risk of bias according to SIGN quality criteria. Only three of the included studies were considered to have a low risk of bias [33,35,37].

Most of the studies (n=8) were focused on lymphatic filariasis alone or together with podoconiosis or onchocerciasis. These define strategies to estimate the prevalence of the diseases or the most common clinical manifestations (hydrocele or lymphoedema). There was one study about NTDs in general [40] and one about sNTDs [10]. Only two studies focused on sNTDs other than lymphatic filariasis. Of these, one was on cutaneous leishmaniasis [38] and one was on leprosy [43]. All studies were conducted in LMICs where sNTDs are endemic, mainly Africa (n=7). Two study areas were in the American continent, and all others were in Africa and Asia. The studies took place in the following 13 different countries: Tanzania (n=4), Ethiopia (n=3), Bangladesh (n=2), Malawi (n=2), Brazil (n=1), Bolivia (n=1), Colombia (n=1), Ghana (n=1), Indonesia (n=1), Nepal (n=1), Nigeria (n=1), Mozambique (n=1), and Peru (n=1). Seven of the studies analyzed interventions in both rural and urban areas, four in rural areas only, and two in urban areas.

**Table 1:** Characteristics of the selected studies.

Reference^a^	Study design	Study population	NTD^b^	Tool	Objective	Intervention	Outcomes	Risk of bias
Sime 2014 [32]	Community-based cross-sectional study	Ethiopia (659 districts) n=129,959 Urban and rural areas	LF^c^ and PDC^d^	LINKS system	To perform integrated mapping of the two diseases	Immunochromatographic card tests of blood samples to detect circulating *Wuchereria bancrofti*. Data collection through questionnaires on a smartphone (GPS function included), and data sent to a cloud server for analysis.	Real-time data collection. Prevalence of lymphoedema (n=8,110) and *Wuchereria bancrofti* (n=139). Budget halved with smartphone-based data collection.	Moderate Selection bias
Luz 2015 [40]	Mixed-methods qualitative study	Southwestern Amazon region (Brazil, Perú, and Bolivia) n=47 (15 medical doctors, 15 researchers, 13 nurses, and four staff) Rural area	NTD	Nu-case	To explore the sustainability of the intervention	Design of Nu-case prototype. Surveys, questionnaires, sketching, and storyboarding to define actors, tasks, needs, and possible design.	High perceived potential of Nu-case in diagnosis, disease monitoring and surveillance, medical records, case notification, and medical research. Low perceived potential in health care management, assisting diagnosis for HCPs^e^ other than medical doctors, and HCP training tools.	High Clustered data analysis with a very small sample size.
Stanton 2015 [33]	Community-based cross-sectional survey	Malawi (Chikwawa district) n=107,331 Ghana (Ahanta West district) n=45,402 Rural area	LF	Measure SMS	To map clinical manifestation of LF (LE^f^ and HYC^g^) To pilot the Measure SMS tool	HSAs^h^ in Malawi and CHWs^i^ in Ghana reported individual LE and HYC case data.	Ghana: prevalence of 17.7 (LE) and 33.0 (HYC) per 10,000. Malawi: prevalence of 76.9 (LE) and 70.5 (HYC) per 10,000. 17% of SMS messages in Malawi and 16% in Ghana contained an error (41% were easy to solve).	Low
Mableson 2017 [39]	Implementation study	Ethiopia, Malawi, Tanzania, Nepal, and Bangladesh n=22 million people Urban and rural areas	LF	Measure SMS-morbidity	To define implementation scenarios for LF mapping	Health workers collect data in a traditional paper-based survey and then send an SMS (two tier) or directly send an SMS (one tier) to a central smartphone connected to a cloud server.	Four potential implementation reporting scenarios: (1) urban, high-endemic setting, two tier (2) rural, high-endemic setting, one tier (3) rural, high-endemic setting, two tier (4) urban and rural, low-endemic setting, one tier	High No methodological details
Mwingira 2017 [34]	Community-based cross-sectional survey	Tanzania (Mtwara Municipal Council) n=108,299 Mainly urban areas	LF	SMS through the GeoPoll platform	To estimate prevalences of HYC and LE	Interactive survey via SMS to randomized selected users.	Response ratio of 15.2%; n=492 (78% male); mean age 20.1 (SD 6.5) years LE signs = 22.2% (95% CI 17.4-24.8) HYC signs = 20.6% (95% CI 16.6-25.0)	High Self-reported data Incentives given to participants Gender and age biased
Mwingira 2017 [35]	Community-based cross-sectional survey	Tanzania (Dar es Salaam) n=5 million Urban area	LF	Measure SMS-morbidity tool	To locate LF patients and estimate prevalence	SMS survey in a three-phase intervention: paper-based data collection checked by a supervisor and sent by SMS to a local server. Data checked and sent to a cloud-based server.	Prevalence of 133.6 per 10,000; n=6.889. Less than 20% of SMS messages had formatting errors. More than 95% of SMS messages were sent to the cloud server.	Low Some implementation challenges
Pedram 2017 [41]	Cross-sectional study	Control (n=77) and infected (n=313) Various countries in Africa, Asia, and America Rural and urban areas	LF and ONC^j^	Smartphone-based microscope	To assess the accuracy of a new diagnostic test	All samples tested with the new test including a portable smartphone-based microscope.	Sensitivity of 71% and specificity around 100% for all tested species.	Moderate Information bias
Karim 2018 [36]	Community-based cross-sectional survey	Bangladesh (high and low endemic districts) n=65 million people at risk Rural and urban areas	LF	Measure SMS	To determine HYC and LE prevalence and develop clinical risk maps for targeted interventions	Paper-based census, active case findings, and summary data per patient sent through the system to a central database. In low endemic districts, case findings via health facility data and confirmed by mHealth^k^ trained professionals sending SMS.	Prevalence of 125.4 per 100,000 in high-endemic districts and 2.4 per 100,000 in low-endemic districts.	Moderate Different methods used mHealth strategy not fully described and validated
Martindale 2018 [37]	Community-based cross-sectional study	Ethiopia (two districts: Hawella Tula and Besa) n=460,722 Rural area	LF and PDC	Measure SMS-morbidity tool	To pilot feasibility and utility for an integrated mapping of the two diseases	Survey conducted by health extension workers (n=59). Clinical cases reported by paper-based standard forms and by SMS.	Paper-based cases reported (n=2.377) SMS method cases reported (n=2.372) *P* value of .94 Prevalence of 64 per 10,000. Cost saving (13.7%) with the SMS method.	Low Potential information bias in the real condition
Mieras 2018 [10]	Development and pilot study	HCP in Nigeria and in Mozambique Rural and urban areas	sNTDs	SkinApp	To support HCP in the early diagnosis of sNTDs in low resource settings	Development of SkinApp with 29 diseases included (six sNTDs) and assessment of user friendliness (semistructured interviews and focus groups). Implementation study	SkinApp is considered a good decision support system.	High No methodological details
Navarro 2018 [38]	Consensus report	Colombia (Tumaco) Rural area	CL^l^	Guaral App	To define challenges and requisites of mHealth tools	Development of Guaral App to be adopted by volunteer community workers for diagnosis and mapping of patients with CL.	Key aspects are: -Sociotechnical context -Systems analysis -Human-centered design	Not applicable
Akoko 2019 [42]	Prospective cohort study	Tanzania (Mtwara and Lindi regions) Rural and urban areas	HYC	WhatsApp platform	To test the efficacy of a mobile platform as an adjunct in supervision and support for nonsurgical clinicians when practicing hydrocelectomy	Didactic and practical training conducted by two experts. Photographs shared though the WhatsApp platform for group discussion, final approval of surgery, on table and postoperative complications.	Fifteen NPCs^m^ trained and able to perform 1337 hydrocelectomies in 1250 patients. Mean procedure duration of 50.2 min (SD 0.24) Complication rate <2.16%	Moderate Small sample of NPCs Selection bias
Rachmani 2019 [43]	Nonrandomized controlled longitudinal observational study	Indonesia (Pekalongan District Java) n=124 control, n=64 intervention group Rural and urban areas	Leprosy	e-leprosy framework	To evaluate the effectiveness of an e-leprosy framework in PHC^n^.	Implementation of an e-leprosy framework for primary health care for 19 months.	Abandon rate of 21%. Increase of 21% in on-time completion and 14.6% in attendance rates.	High Nonrandomization Sample selection bias

^a^Studies are in chronological order and then alphabetical order of first author.

^b^NTD: neglected tropical disease.

^c^LF: lymphatic filariasis.

^d^PDC: podoconiosis.

^e^HCP: health care professional.

^f^LE: lymphoedema.

^g^HYC: hydrocele.

^h^HSA: salaried health surveillance assistant.

^i^CHW: community health worker.

^j^ONC: onchocerciasis.

^k^mHealth: mobile health.

^l^CL: cutaneous leishmaniasis.

^m^NPC: nonphysician clinician.

^n^PHC: primary healthcare.

### Elements Included in the mHealth Interventions

Only eight different mHealth interventions were identified (Table 2). Three were based on sending SMS text messages between health workers and a central web system [33-35,37,39,41,43]. Text messaging is an easy technology to use and, as these studies show, is useful for motivating patients to complete treatments. Health care services can be equipped with systems providing automated text message reminders for disease control [43]. The Measure SMS and Measure SMS-morbidity strategies have been tested and implemented in several different contexts. The ability to view and assess the quality of patient identification data in real time is a great asset. Enabling information on morbidity burden to be known almost instantaneously, as opposed to having to wait for the collation and digitization of paper forms, also facilitates more efficient provision of necessary care [35].

**Table 2:** Elements and challenges described in the mobile health interventions of the selected studies.

Tool Name	Elements included				Challenges identified	References
	SMS	Web	App	Cloud server		
e-Leprosy framework	+	+	−	−	Cost-benefit analysis Need for a comprehensive strategy Perceived usefulness of the technology Perceived ease of use of the technology No internet connection in remote rural areas	Rachmani 2019 [43]
GeoPoll and SMS	+	+	−	−	No challenges identified in the study	Mwingira 2017 [34]
Guaral App	−	−	+	+	Sociotechnical context Human-centered design Iterative design Usability Technical concerns	Navarro 2018 [38]
LINKS system	−	−	+	+	Data ownership Lack of technical expertise Cost of smartphones HCP^a^ perceptions about digital data collection Batteries running out of charge Lack of network	Sime 2014 [32]
Measure SMS and Measure SMS-morbidity	+	+	−	+	Data accuracy HCP training Technological barriers Availability of mobile phones Cost Poor network coverage Battery supply Feasibility Health system structure and organizational change	Stanton 2015 [33] Karim 2019 [36] Mableson 2017 [39] Mwingira 2017 [35] Martindale 2018 [37]
Nu-case	−	−	+	+	Heterogeneity of linguistic cultural backgrounds Potential legal issues Network signal coverage Durability and battery life Image and data quality Empowerment of local health workers	Luz 2015 [40]
SkinApp	−	−	+	+	Technical requirements Cost Need of context-specific adaptation (language, culture, and epidemiological situation)	Mieras 2018 [10]
WhatsApp	−	−	+	−	Data privacy Highly time consuming Work required on mentor and mentee relationship Internet connection requirement	Akoko 2019 [42]

^a^HCP: health care professional.

Five interventions were conducted through apps and cloud servers. One utilized the commercially available WhatsApp app [42], and the others used either very simple ad-hoc developed apps able to collect data and send to a server [32] or apps that included a wider range of elements and took a more comprehensive approach, for example, Guaral App [38], Nu-case [40], and SkinApp [10]. Only Guaral App and SkinApp are available from the app stores (Table 3).

**Table 3:** Characteristics of apps for skin neglected tropical diseases available from app stores in alphabetical order.

Name (date last updated)	Marketplace (number of downloads)	Objective	Target user	Disease
EndNTDs App (October 2017)	Google Play Store (+100)	To create a community of champions who are at the forefront in the fight against NTDs^a^ in Zimbabwe, Africa, and globally	HCP^b^	NTDs (including skin NTDs)
Guaral RPC (February 2020)	Google Play Store (+10)	To improve early diagnosis	HCP	CL^c^
Lepra Reaction Basic management guide (October 2017)	Google Play Store (+100)	To conduct classification and quantification of severity to plan for appropriate clinical management	HCP	Leprosy
LymEX (July 2019 Google Play Store and January 2020 Apple App Store)	Google Play Store (+50); Apple App Store	To improve self-care	Patients	Lymphoedema
LymVol (September 2019)	Google Play Store (+10)	To measure and calculate limb volume of those affected by edema	HCP	Lymphoedema
Recognize Hydrocele disease (September 2019)	Google Play Store (+500)	To improve information about causes, treatment, and complications	Patients	Hydrocele
Recognize scabies (September 2019)	Google Play Store (+100)	To improve knowledge about the disease	Patients	Scabies
Scabies disease (December 2017)	Google Play Store (+500)	To improve clinical management of the disease	HCP	Scabies
Scabies Disease: Treatment (October 2019)	Google Play Store (+100)	To manage symptoms and treatment	Patients	Scabies
SkinApp (October 2019)	Google Play Store (+10); Apple App Store	To act as a diagnostic tool and source of information on signs, symptoms, and therapy	HCP	NTD- and HIV-related skin diseases
Skin NTDs App (July 2020)	Google Play Store (+60); Apple App Store	To diagnose and identify signs and symptoms of sNTDs through their visible characteristics	HCP	Skin NTDs
Task Force Tropical Data (August 2016)	Google Play Store (+1000)	To collect data	Task Force for Global Health members	NTDs (including skin NTDs)
Tropical Diseases (August 2019)	Google Play Store (+1000)	To provide a detailed overview of the etiology, pathophysiology, epidemiology, diagnosis, and treatment of tropical diseases	HCP	TDs^d^ including CL, BU^e^, and leprosy
Tropical Diseases (December 2019)	Google Play Store (+100)	To provide a detailed overview of the cause, diagnosis, prognosis, risk factors, prevention, and treatment of the stated diseases	HCP	TDs including CL, BU, and leprosy
WIDP (January 2020)	Google Play Store (+10)	To control the epidemiological situation	HCP and WHO^f^ staff	Yaws and BU

^a^NTD: neglected tropical disease.

^b^HCP: health care professional.

^c^CL: cutaneous leishmaniasis.

^d^TD: tropical disease.

^e^BU: Buruli ulcer.

^f^WHO: World Health Organization.

Results showed that mHealth strategies do achieve their primary goals, but many challenges require consideration. Both SMS and app interventions face (1) technological, (2) legal, (3) final user, and (4) organizational issues (Table 2) as follows:

- The main technological issues are poor network coverage or no internet connection in specific areas, batteries running out of charge, poor data and image quality, and lack of technical requirements.
- Legal issues, including data privacy and ownership, are important concerns that must be resolved.
- Final users can be health care professionals, patients, and citizens, who all require adequate digital literacy and to feel more empowered.
- Final users need to perceive the technology as possessing a high level of usefulness and ease of use.
- The organizational challenges most often identified relate to resources (cost, time, and availability of smartphones), implementation planning, cultural and language barriers, and the need for user features to be fully completed when implementing comprehensive mHealth strategies.

mHealth interventions using apps also involve extra challenges in the development period, such as consideration of the sociotechnical context, and the need for human-centered and iterative design to improve usability, feasibility, and user experience.

### Selection of Apps

Fifteen apps were identified in the Google Play Store, with three of these also available in the Apple App Store. Most of the apps (11/15, 73%) were targeted at health care professionals, with only four targeted at patients. The number of app downloads can be taken as a proxy indicator of use. However, while Google Play Store comprehensively provides this information, the Apple App Store provides it to the app developer only. Google Play Store data revealed that only two of the apps had more than 1000 downloads. These were Task Force Tropical Data, which is mainly used to collect data, and Tropical Diseases, which focuses on various tropical diseases but only provides information on three sNTDs (Buruli ulcer, cutaneous leishmaniasis, and leprosy). Most of the apps have recently been updated or uploaded, with only four not updated over the last 12 months. The apps addressed scabies (3/15, 20%), lymphatic filariasis (3/15, 20%), cutaneous leishmaniasis (1/15, 7%), leprosy (1/15, 7%), yaws and Buruli ulcer (1/15, 7%), tropical diseases including more than one sNTDs (3/15, 20%), and NTDs including sNTDs (2/15, 13%). Only 1 (7%) app was related to the clinical management of sNTDs (Table 3).

## Discussion

### Principal Findings

We reviewed the literature on the evidence for mobile apps and have providing a set of recommendations for the future development and implementation of such tools. Our review pinpointed 13 studies dealing with mobile health interventions for managing sNTDs in LMICs. A descriptive evidence synthesis showed that most of the studies had a high or moderate risk of bias according to SIGN quality criteria, and only three were considered to have a low risk of bias. Only two studies focused on sNTDs other than lymphatic filariasis, one focused on cutaneous leishmaniasis, and one focused on leprosy.

In addition, eight different mHealth interventions were identified, of which three were based on texting by SMS and five were conducted through apps and cloud servers. Moreover, we acknowledged 15 apps available in app stores. Most of the apps (11/15, 73%) were targeted at health care professionals, with only four targeted at patients. Results showed that mHealth strategies do achieve their primary goals, but many challenges require consideration. All mHealth interventions face technological, legal, final user, and organizational issues.

The disease most targeted in mHealth interventions for sNTDs is lymphatic filariasis. The Global Program to Eliminate Lymphatic Filariasis as a public health problem was launched in 2000, and the global elimination goal of 2020 established in 2012 was probably a key factor in driving the Ministry of Health, the WHO, and implementing partners to scale up surveillance and morbidity management activities for this disease. There is an urgent need for a rapid and adaptable tool to gather patient estimates in order for national programs to appropriately forecast, plan, and deliver a basic package of care to those suffering from the disabling and debilitating clinical manifestations of lymphatic filariasis in an affordable manner [39]. Three of the apps on the market address scabies, but as no trials have been identified, there is a lack of existing evidence about mHealth interventions to manage this disease. Other sNTDs, such as cutaneous leishmaniasis and leprosy, have been managed with pilot interventions through mHealth strategies. It is interesting to note that a number of trials and apps are focused on an integrated approach to the various sNTDs. The most recent app developed by WHO aims to provide support for diagnosis by using an automated algorithm to provide potential conditions from the selection of sNTD signs and symptoms.

The provision of health care services to populations in remote regions, often dispersed over large geographical areas, has long been considered a challenging issue from the perspective of health systems management [44]. Telemedicine approaches have been employed in some places for remote assistance for diagnosis and treatment, with a degree of success [45], but neglected diseases, and therefore sNTDs, require more than the usual functionality of telemedicine systems [40]. mHealth interventions in high-income countries are focused on apps, but as sNTDs mainly affect populations with low resources in LMICs, most of the interventions tested so far were based on SMS text messaging, and only a few app strategies have been attempted. Using text messages to change patient behavior and achieve targeted health outcomes also requires a comprehensive strategy but demands fewer resources. Studies in Indonesia have addressed the apparent difficulties in leprosy control programs and how a health information system could assist, and concluded that SMS text messaging and web-based applications could be good strategies for implementing continuous monitoring and recording of patients [43]. The advantage of SMS is “internet-less” connectivity, and it allows fast transmission of surveillance data, whereas store-and-forward teledermatology represents an ideal approach to assess skin lesions and provide long-distance support to individual diagnosis because images are shared and can be assessed. The cost-effectiveness of store-and-forward teledermatology increases when patients are required to travel farther distances to access dermatology services. SMS and store-and-forward teledermatology are complementary and contribute to improve the epidemiological and clinical management of sNTDs.

By bringing essential central health services closer to peripheral areas, innovative technological methods like telemedicine, mHealth apps, and SMS text messages can help to bridge the gap between the burden of skin diseases and the lack of capable staff in resource-poor settings [10]. mHealth interventions enable rapid data collection, easy monitoring and supervision of data reporting, better management of sNTDs, and efficient provision of necessary care. They also appear to be lower cost strategies than paper-based forms that require more human and financial resources. The four studies that conducted cost analyses [33,35-37] did not have sufficient robust data, but all concluded that mHealth interventions are less costly than paper-based epidemiological mapping. By managing and taking full ownership of data and the implementation process, local teams will be empowered, and the establishment of valuable data management and health surveillance capacities within the local team and at the country level will empower national health systems [35].

### Recommendations Based on the Existing Evidence

Based on evidence presented in the studies identified in this systematic review, the following eight recommendations will enable sNTD mHealth-based interventions to move forward from innovation to implementation:

1. No one should be left behind. Patients from all regions must be targeted to benefit from the proposed interventions. This requires translation of the tools into several languages. At least into Portuguese and Spanish in the Americas, and English, French, and Portuguese in Africa.
2. Users must be empowered. Final users of the interventions (health care professionals and/or patients) should receive sufficient training to improve their digital literacy and make appropriate use of the tools.
3. Complexity must be addressed. The adoption of digital health technology is a complicated process that needs to be thoroughly considered before and during implementation.
4. Utility and simplicity must be perceived. Health care professionals, patients, and healthy citizens should grasp the utility and user friendliness of the proposed technology, in order for these two elements to become enablers and not barriers.
5. Technological requirements must be considered from the very outset. The availability of smartphones and potential problems with electricity or internet networks must be addressed as part of a comprehensive strategy with a specific aim.
6. A long-term mHealth platform must be put in place. The success of an mHealth intervention depends on the existence of an mHealth platform to not only facilitate adoption of the tool, but also guarantee sustained effective use.
7. Two-tiered processes are required for improvement. In the first stages of implementing an mHealth intervention, it is important to have two-tiered processes to refine and optimize the process in an iterative way.
8. The tool must respond to needs. Interventions are embedded in a specific health service; therefore, additional tools must be considered according to the need.

### Limitations

One of the main limitations of this review is publication bias. References from other sources, such as conferences and meetings, have not been included. Although the number of scientific journals that publish mHealth-related articles has increased in recent years, there is a lot of grey literature surrounding this field that we may have missed. Moreover, the heterogeneity of interventions and populations has made it difficult to synthesize results, and consequently, findings need to be considered with caution. Most studies in this review were conducted in East Africa, and the total number of countries represents 9% of those with NTDs worldwide. This limits the extrapolation of the results.

### Conclusions

The potential for mHealth interventions to improve the epidemiological and clinical management of sNTDs is yet to be reached. There are very few good quality studies and tools, and most of these have methodological limitations, leaving considerable room for improvement. The majority of sNTDs are not addressed by any mHealth tool. This research has enabled us to identify eight recommendations for the future development and implementation of mHealth interventions for managing sNTDs in LMICs.

## Appendix

Multimedia Appendix 1 Search strategy.

## Abbreviations

LMIC: low- and middle-income country
mHealth: mobile health
NTD: neglected tropical disease
SIGN: Scottish Intercollegiate Guidelines Network
sNTD: skin neglected tropical disease
WHO: World Health Organization
