# Supplementary material for: Mobile Health Strategies to Tackle Skin Neglected Tropical Diseases With Recommendations From Innovative Experiences: Systematic Review
Source: JMIR Mhealth Uhealth. 2020 Dec 31;8(12):e22478. doi: 10.2196/22478 (PMC7808891; doi:10.2196/22478)
Supplement: Multimedia Appendix 1 [file mhealth_v8i12e22478_app1.docx]

mHealth strategies to tackle skin NTDs: recommendations from innovative experiences

###

### **Studies search strategy**

### **PubMed**

###

### (filarial lymphoedema OR scabies) OR onchocerc* OR onchocerca OR mycetoma OR lymphatic filariasis OR leprosy OR yaws OR buruli ulcer OR buruli OR leishmania OR cutaneous leishmaniasis OR visceral leishmaniasis OR neglected tropical diseases) AND ("mhealth" OR “mobile app” OR “SMS” OR “health app” OR "smartphones")

### **Scopus**

TITLE-ABS-KEY ( ( mhealth OR "health apps" OR "mobile apps" OR sms OR smartphones ) AND ( mycetoma OR "neglected disease*" OR leishmanias* OR "filarial lymphoedema" OR scabies OR onchocerc* OR leprosy OR "buruli ulcer" OR yaws )

### **Apps search strategy**

The terms used in both Apple Store and Google Play app stores were:

- NTD
- Neglected Tropical Diseases
- Skin Tropical Diseases
- Buruli ulcer
- Cutaneous leishmaniasis
- Post-kala-azar dermal leishmaniasis
- Leprosy
- Lymphatic filariasis (lymphoedema and hydrocele)
- Mycetoma
- Onchocerciasis
- Scabies
- Yaws
- Fungal diseases
